# Associated Factors of Acute Chest Syndrome in Children with Sickle Cell Disease in French Guiana

**DOI:** 10.1155/2014/213681

**Published:** 2014-01-19

**Authors:** Narcisse Elenga, Emma Cuadro, Élise Martin, Nicole Cohen-Addad, Thierry Basset

**Affiliations:** ^1^Pediatric Unit, Cayenne Medical Center, Andrée Rosemon Hospital, Rue des Flamboyants, BP 6006, 97306 Cayenne Cedex, French Guiana; ^2^Pediatric Unit, Kourou Medical Center, Avenue des îles, BP 703, 97310 Kourou, French Guiana

## Abstract

A matched case-control study was performed in order to identify some associated factors for ACS or to confirm the published data. Controls were children hospitalized during the same period for pain crisis who did not develop an ACS during hospitalization. Between January 2006 and October 2010, there were 24 episodes of ACS distributed among 19 patients (8 girls and 11 boys). The median age was 7.5 years (range: 3 to 17 years) for the cases and 7 years (range: 3–18 years) for the controls. Four cases and 11 controls were treated with hydroxyurea (HU). In 75% of the cases, the ACS had arisen 24–72 hours following admission. The independent factors associated with ACS were average Hb rate <8 g/dL (OR = 4.96, 95% CI = 1.29–27.34, and *P* = 0.04), annual number of hospitalizations >3 (OR = 5.44, 95% CI = 3.59–8.21, and *P* = 0.003), average length of hospitalization >7 days (OR = 3.69, 95% CI = 3.59–8.21, and *P* = 0.003), and a pathological transthoracic echocardiography (TTE) (OR = 13.77, 95% CI = 2.07–91.46, and *P* = 0.003). Although the retrospective design and small sample size are weaknesses of the present study, these results are consistent with those of previous studies and allowed identifying associated factors such as a pathological TTE.

Sickle cell disease (SCD) is a major public health concern in French Guiana, a French region with 230,000 inhabitants located in South America [1]. The incidence of major SCD from birth screening is 1/227, and the overall frequency of AS carriers is 10% [2]. The major SCD groups include the three main genetic forms: hemoglobin (Hb) SS (68%), Hb SC (25%), and S*β* thalassemia (7%). The acute chest syndrome (ACS) is a complication of SCD characterized by pleuritic chest pain, fever, rales on lung auscultation, and pulmonary infiltrates on chest X-ray [3]. It is the most frequent cause of mortality in children with SCD [3–8]. In 1979, Charache et al. first suggested using the term acute chest disease (ACD) for this complication, acknowledging the difficulties in determining its pathogenesis [9].

We report here the results of a case-control study of risk factors for ACS in children with SCD in French Guiana, in order to find some associated factors for ACS or to confirm the published data. We hypothesized that HbSS, age, high Hb level, and high steady-state leukocyte count could be risk factors for ACS. This matched case-control study concerned all cases of ACS hospitalized in the pediatric unit in French Guiana from 2006 to 2010. The cases were children hospitalized between January 2006 and October 2010 for pain crisis and who developed an ACS. The controls were children hospitalized during the same period for pain crisis and who did not develop an ACS during hospitalization. Each episode of ACS was matched on age, gender, and year of diagnosis.

The transthoracic echocardiography (TTE) was performed by a single pediatrician cardiologist, at baseline when the child was in a healthy state, during the annual evaluation. Patients with a pathological TTE were followed every six months by the same pediatrician cardiologist. All the TTE were obtained at true baseline and not during admissions. These TTE showed the following anatomical pathologies: an enlargement of the left heart chambers associated with an elevation in blood volume in seven cases and two controls and elevation in left ventricular myocardial indices in two cases and two controls. The Commission Nationale Informatique et Libertés approved our data collection. The factors associated with ACS were analyzed by logistic regression based on odds ratios (OR). For all tests performed, a *P* value of 0.05 or less was considered as statistically significant. The data were entered into Microsoft Excel 2007 and analyzed using R.2.10.0 (R project, CRAN R 2.10.0 version 2010) statistical software. All the factors numbered in Table 1 were included in this analysis. We included in our final model the covariates that were associated with the outcome in the univariate analysis and other factors associated with ACS, according to the literature.

Between January 2006 and October 2010, there were 24 episodes of ACS distributed among 19 patients (8 girls and 11 boys). One patient developed 3 events of ACS; 3 presented 2 events; and 15 patients presented 1 event of ACS. The median age was 7.5 years (range: 3 to 17 years) for the cases and 7 years (range: 3–18 years) for the controls (Mann-Whitney test). These two groups did not differ in age.

Four cases and 11 controls were treated with hydroxyurea (HU). The results are shown in Table 1. Among the cases, there were 20 HbSS, 3 HbS*β*° thalassemia, and 1 HbSC. Among the controls, there were 45 HbSS, 12 HbS*β*° thalassemia, 10 HbS*β*+ thalassemia, and 9 HbSC. The more frequent hospitalization, longer hospital stays, lower Hb, higher % HbS, and earlier presentation in the cases may be explained, at least in part, by the difference in genotype and the severity of sickle cell anaemia.

In 75% of the cases, the ACS had arisen 24–72 hours following admission. All patients received rehydration, oxygen therapy, and pulmonary physiotherapy using stress spirometry and triple antibiotic therapy (cefotaxime, aminoglycoside, and a macrolide). In 16 cases, patients received a single red blood cell transfusion and in six other cases, the red blood cell transfusion was followed by a partial exchange transfusion (removing the patient's own blood and replacing it with 0.9% NaCl volume for volume, which was followed by a red blood cell transfusion). The target haemoglobin S value was under 30% and haemoglobin level 80–90 g/L, and in any of the cases, we obtained them. The more frequent hospitalization, longer hospital stays, lower Hb, higher % HbS, and earlier presentation in the cases may be explained, at least in part, by the difference in genotype and the severity of sickle cell anaemia. Two cases died. The cases who died did not receive a transfusion. The first was a 6-year-old girl in severe vasoocclusive crisis with fever and severe anemia (Hb of 4 g/dL), who presented 8 hours later with a frank ACS and whom endotracheal intubation was unsuccessful. The second was a 4-year-old girl also with frank ACS and bilateral pleural effusion, who died despite successful endotracheal intubation. The two cases who died had received a single dose of ceftriaxone. The proportion of death was high and due in part to low access to care in foreign patients. Although health is free of charge in French Guiana, the prevention is complicated by the fact that a number of persons are illiterate or do not understand the language. However, the health priorities of immigrants are often overridden by daily struggles to obtain food, shelter, and papers. Due to the low power of our study, certain factors such as lower Hb, higher % HbS, and earlier presentation in the cases as well as the transcranial Doppler, that were significant in bivariate analysis, were not statistically significant in multivariate analysis. However, the effect of including several episodes in single individuals was not analysed because of the small sample of our study.

Annual number of hospitalizations >3 (OR = 5.44, 95% CI = 3.59–8.21, and  *P* = 0.003), average length of hospitalization >7 days (OR = 3.69, 95% CI = 3.59–8.21, and *P* = 0.003), average Hb rate <8 g/dL (OR = 4.96, 95% CI = 1.29–27.34, and *P* = 0.04), and a pathological TTE (OR = 13.77, 95% CI = 2.07–91.46, and *P* = 0.003) were independent associated factors for ACS. The TTE was performed to detect any abnormalities such as left heart chambers abnormalities and intracardiac shunts, including foramen ovale. According to a few studies [10], intracardiac shunting could be a risk factor for stroke in children with SCD because it predisposes to thrombosis and elevations of right heart pressure, which could promote paradoxical embolization across an intracardiac shunt. In our study, a pathological TTE was a strong factor associated with ACS. However, the role of cardiac abnormalities as associated factors for children with ACS was unknown. Defining the role of intracardiac shunting in pediatric ACS will require controlled studies with unified detection methods in stratified populations. HU is efficacious in children and adults with SCD, with an increase in Hb F%, reduction in hospitalizations and pain crises, and prevention of new episodes of ACS. However, in our study, because of its low power, this variable did not appear as a protective factor. Although the retrospective design and small sample size are weaknesses of the present study, our results are consistent with those of previous studies [3, 4, 9]. This study also allowed us to identify associated factors such as pathological TTE. Possible preventative measures consist of earlier use of red blood cell transfusion and/or early use of HU. The role of cardiac abnormalities in ACS deserves to be clarified by other studies.

## Figures and Tables

**Table 1:** Case and control description and bivariate and multivariate analysis*.

Variables	Cases (%)	Controls (%)	Bivariate analysis		Multivariate analysis	
			Crude OR (95% CI)	*P*	Adjusted OR (95% CI)	*P*
Age (years)						
0–4	2 (8)	25 (33)	1			
5–9	14 (59)	21 (27)	0.12 (0.02, 0.59)	0.009		
10–14	6 (25)	15 (20)	0.2 (0.04, 1.12)	0.07		
15–19	2 (8)	15 (20)	0.6 (0.08, 4.72)	0.63		
Sex						
M	13 (54)	43 (57)	1			
F	11 (46)	33 (43)	1.1 (0.44, 2.77)	0.84		
Type of haemoglobin						
SS	20 (83)	45 (59)	1			
S*β* thal or SC	4 (17)	31 (41)	0.29 (0.09, 0.93)	0.038		
History of treatment by hydroxyurea						
Yes	4 (17)	11 (14)	1			
No	20 (83)	65 (86)	0.85 (0.24, 2.95)	0.79		
Duration of hospitalization (days)						
>7	14 (58)	12 (16)	4.23 (2.64, 6.3)	<0.01	**3.69 (2.30–5.56)**	<0.01
0–7	10 (42)	64 (84)	1			
Age during the first symptoms						
1 year	5 (21)	34 (45)	1			
Before 1 year	19 (79)	42 (55)	3.08 (1.04, 9.09)	0.04		
History of >3 annual hospitalisations						
Yes	19 (79)	26 (34)	7.31 (2.45, 21.8)	<0.01	**5.44 (3.59–8.21) **	*0.003 *
No	5 (21)	50 (66)	1			
Basic haemoglobin level						
>8 g/dL	3 (12)	31 (41)	1			
0–8 g/dL	21 (88)	45 (59)	4.82 (1.32, 17.58)	0.15	**4.96 (** **1.29–27.34)**	*0.04 *
Basic S haemoglobin level						
≥80%	20 (83)	39 (51)	1			
<80%	4 (17)	37 (49)	0.21 (0.07, 0.68)	0.009		
Transthoracic echocardiography						
Normal	15 (63)	70 (95)	1			
Abnormal	9 (37)	4 (5)	10.5 (2.85, 38.65)	<0.001	**13.77 (2.07–91.46)**	***0.003***
Transcranial echo-Doppler						
Normal	19 (79)	72 (97)	1			
Abnormal	5 (21)	2 (3)	9.47 (1.7, 52.69)	0.01		

*Obtained using conditional logistic regression with indicator variables for nonbinary variables.

Bold fonts: OR (95% CI) of multivariate analysis.

Italic fonts: *P*-value of multivariate analysis.
